# The perception of pre and postoperative pain management among recently operated patients in Saudi Arabia

**DOI:** 10.3389/fpubh.2026.1773870

**Published:** 2026-04-22

**Authors:** Maram H. Alshareef, Abduljalil A. Alsafi, Khalid F. Salaemae, Fahad A. Alshumrani, Mousa M. Alshumrani, Feras B. Kofiah, Mulham M. Hariri, Khalid Basamih, Majd Almech, Mohammed Shaikhomer, Asim M. Alshanberi, Safaa M. Alsanosi

**Affiliations:** 1 Department of Community Medicine and Pilgrims Health Care, Faculty of Medicine, Umm Al-Qura University, Makkah, Saudi Arabia; 2 Faculty of Medicine, Umm Al-Qura University, Makkah, Saudi Arabia; 3 General Medicine Practice Program, Batterjee Medical College, Jeddah, Saudi Arabia; 4 Department of Internal Medicine, Faculty of Medicine, King Abdulaziz University, Jeddah, Saudi Arabia; 5 Department of Pharmacology and Toxicology, Faculty of Medicine, Umm Al-Qura University, Makkah, Saudi Arabia

**Keywords:** management, patient, postoperative pain, preoperative, recovery

## Abstract

**Background:**

Despite advances in surgical and anesthetic techniques, postoperative pain remains a prevalent and often inadequately managed clinical issue. Poorly controlled postoperative pain is associated with delayed recovery, increased morbidity, and reduced patient satisfaction. In Saudi Arabia, there is limited evidence on real-world pain management practices and their outcomes.

**Aim:**

This study aims to evaluate the effect of pain management strategies on postoperative recovery, providing evidence to guide patient-centered and efficient pain control protocols.

**Methods:**

A cross-sectional study was conducted in Makkah between July and September 2025. Participants included adult patients (aged 15–75) who underwent major surgery affecting mobility. Data were collected via a structured questionnaire incorporating the APS-POQ-R tool, covering demographics, pre- and postoperative pain experience, management practices, and patient perceptions. Descriptive and inferential statistical analyses were conducted using SPSS version 28. A *p*-value of <0.05 was considered statistically significant.

**Results:**

A total of 207 participants were included. Almost half were females (58.9%) and aged 30–49 years (47.8%). Abdominal surgeries were most common (43.1%). Most participants experienced moderate to severe pain within 24 h post-surgery, though pain intensity decreased by the end of the first week. Only 64.7% received preoperative counseling on pain control, and 31.4% were involved in analgesic decision-making. While 76.8% were prescribed home analgesics, 33.8% reported adverse side effects. Satisfaction with pain management was significantly associated with marital status, nationality, type of hospital, mode of anesthesia, timeliness of analgesia, and the effectiveness of home medication (*p* = 0.001). Patients who received both pharmacological and non-pharmacological education, as well as those who felt well-prepared for postoperative pain, reported significantly higher satisfaction (*p* = 0.001).

**Conclusion:**

Effective pain management significantly improved recovery, mobility, and satisfaction. However, gaps remain in patient education, shared decision-making, and non-drug interventions. Enhancing preoperative counseling, multimodal pain control, and patient-provider communication is recommended to optimize postoperative outcomes in Saudi healthcare settings.

## Introduction

Postoperative pain continues to pose a significant clinical challenge, affecting a substantial number of patients following surgical interventions. Effective management remains essential, as inadequate pain control is associated with delayed functional recovery, extended hospital stays, increased risk of postoperative complications, decreased patient satisfaction, and a greater likelihood of developing chronic post-surgical pain (1, 2). Optimal control of postoperative pain is now recognized as a fundamental element of modern perioperative care, promoting enhanced recovery, reducing complications, and improving overall surgical outcomes (3). Despite remarkable progress in surgical and anesthetic techniques, over 50% of patients still report moderate to severe postoperative pain (4), which may increase morbidity and contribute to the transition from acute to chronic pain (5). Inadequately managed acute pain can also impair physical function, hinder mobility, and negatively influence psychological well-being, thereby delaying recovery (1). The adoption of multimodal analgesia has revolutionized postoperative pain management by integrating multiple classes of analgesics and complementary techniques to act on distinct pain pathways, effectively reducing reliance on opioids and minimizing the related adverse effects (6). This strategy not only enhances the quality of analgesia but also promotes early ambulation and functional recovery, both of which are essential for minimizing postoperative complications and optimizing patient outcomes (7). Engaging patients in discussions about perioperative and postoperative pain management options fosters shared decision-making, enhances pain control, improves the overall patient experience, and promotes faster recovery and rehabilitation (6, 8). Moreover, incorporating patient participation in pain management decision-making enhances satisfaction and fosters a sense of autonomy and trust in care (9). However, a considerable proportion of patients continue to experience inadequate pain control, largely due to insufficient knowledge and understanding of the available pain management options (10, 11). Moreover, many patients report unpleasant side effects from analgesic medications, which may further hinder treatment adherence and negatively impact overall satisfaction with postoperative care (12). Research has demonstrated that patient education plays a crucial role in enhancing postoperative pain control and satisfaction (11). In Saudi Arabia, there are limitations in the published research examining the real-world effectiveness of postoperative pain management strategies in hospitals. Understanding how various pain control practices influence key recovery indicators—such as pain intensity, early mobilization, hospital length of stay, and postoperative complications—is essential for enhancing the quality of perioperative care and improving patient outcomes (13). This study aims to evaluate the effect of pain management strategies on postoperative recovery, providing evidence to guide patient-centered and efficient pain control protocols.

## Method

### Ethical approval

Bioethical approval was obtained for this study from the institutional research ethics board of Umm Al Qura University with code (HAPO-02-K-012-2025-06-2803).

### Study design

This is a cross-sectional study conducted in Makkah city between July and September 2025 among the general population. We included stable, healthy patients who had major surgery affecting mobility, and excluded people with major comorbidities, the older adults with cognitive impairment, people under 15 or over 75 years of age, and patients who only had minor surgery that did not affect mobility.

### Questionnaire tool and data collection

The study utilized the Revised American Pain Society Patient Outcome Questionnaire (APS-POQ-R) to assess patients’ postoperative pain experiences, including pain intensity, functional interference, emotional responses, and satisfaction with pain management (14, 15). To ensure linguistic and cultural suitability for the Arabic-speaking population, the questionnaire underwent a structured translation and adaptation process following established cross-cultural research guidelines.

First, the original English version of the APS-POQ-R was independently forward translated into Arabic by two bilingual translators with medical backgrounds. The two translated versions were compared and synthesized into a single preliminary Arabic version after resolving minor discrepancies. Subsequently, back-translation into English was performed by an independent bilingual translator who was blinded to the original questionnaire, allowing verification of conceptual and semantic equivalence between the translated and original versions.

To establish content validity, the Arabic version was reviewed by an expert panel consisting of three specialists in pharmacology, anesthesia, and pain medicine. The experts evaluated the clarity, cultural relevance, and appropriateness of the questionnaire items for the Saudi patient population. Based on their recommendations, minor wording modifications were made to improve clarity and contextual suitability.

The pre-final Arabic questionnaire was then pilot tested among 20 postoperative patients who met the study eligibility criteria to assess comprehension, clarity, and feasibility. Participants reported that the questionnaire was clear and easy to understand, and only minor linguistic adjustments were required. Data from the pilot study were not included in the final analysis.

The internal consistency reliability of the questionnaire was assessed using Cronbach’s alpha coefficient. The overall scale demonstrated good internal consistency (Cronbach’s *α* = 0.87). Reliability analysis of the major domains also showed acceptable to good internal consistency, with Cronbach’s alpha values of 0.84 for pain severity items, 0.81 for functional interference, 0.79 for emotional impact, and 0.85 for communication and satisfaction items, indicating that the instrument was reliable for assessing postoperative pain outcomes in the study population.

. The questionnaire included demographic information, as well as five main sections.

- The first section included information related to the surgery, including the type and name of the surgery, the area of the body that underwent surgery (e.g., bones, abdomen, chest, or pelvis), whether they had a companion after the surgery, and the medications they received during and after the operation.
- The second section assessed participants’ pain experience in the preoperative phases, including assessing awareness of pain management options, concerns about pain, and postoperative pain preparedness.
- The third section assessed participants’ experience of pain during their hospital stay, including questions about the type of anesthesia, the timing and intensity of pain after surgery, communication with healthcare providers, participation in pain management decisions, and the side effects of painkillers.
- The fourth section included an assessment of medication adherence after hospital discharge, pain relief effectiveness, and average pain level during the first week, impact of pain on mobility, daily activities and sleep, use of non-drug methods, and satisfaction with pain management.
- The fifth section assesses participants’ general perceptions and offers an opportunity to give suggestions. Participants were asked about their general perception of pain control after surgery, whether effective pain management had improved their mobility and recovery, and were given an open question to offer suggestions for improving postoperative pain care.

All questions were either closed (multiple choice, Likert-type, or numerical rating scale 0–10) or open-ended to capture qualitative feedback. The survey was distributed electronically via Google Forms through medical interns in the Makkah community, ensuring agreement before access for participants.

Reliability values for the study questionnaire.

**Table:** 

Domain	Cronbach α
Pain severity items	0.84
Functional interference	0.81
Emotional impact	0.79
Communication and satisfaction	0.85
Overall scale	0.87

### Data analysis

All statistical analyses were performed using IBM SPSS Statistics for Windows, Version 28.0 (IBM Corp., Armonk, NY, United States). Descriptive statistics were used to summarize the socio-demographic characteristics, surgical details, pain experiences, pain management practices, and patient satisfaction. Categorical variables were presented as frequencies and percentages. Bivariate analyses were conducted to examine the associations between participants’ satisfaction with pain management and potential influencing factors, including socio-demographic characteristics, type of hospital, type of anesthesia, preoperative counseling, pain severity, and involvement in pain management decisions. The Chi-square test (*χ*^2^) was primarily used for comparisons of categorical variables. For cells with expected counts less than five, Fisher’s exact test or Exact Probability test was applied. A *p*-value of <0.05 was considered statistically significant.

## Results

A total of 207 participants were included in the study. Most participants were aged 30–49 years (*n* = 99; 47.8%). Females constituted a larger proportion of the sample (*n* = 122; 58.9%). Regarding marital status, about half of the participants were married (*n* = 104; 50.2%), whereas 50 (24.2%) were single. The majority were Saudi nationals (*n* = 144; 69.6%). Regarding educational level, most respondents had a diploma or university degree (*n* = 132; 63.8%). In terms of occupational status, 82 (39.6%) were employed. More than half of the participants reported having a chronic disease (*n* = 110; 53.1%). Among those with disease, diabetes mellitus and hypertension were the most commonly reported, each affecting 18 (16.4%) participants (Table 1).

**Table 1:** Bio-demographic characteristics of the study participants (*N* = 207).

Bio-demographic data	No	%
Age in years		
18–29	71	34.3%
30–49	99	47.8%
>50	37	17.9%
Gender		
Male	85	41.1%
Female	122	58.9%
Marital status		
Single	50	24.2%
Married	104	50.2%
Divorced/widow	53	25.6%
Nationality		
Saudi	144	69.6%
Non-Saudi	63	30.4%
Educational level		
Basic education	31	15.0%
Diplôme, University	132	63.8%
Post-graduate	44	21.3%
Work status		
Not working	66	31.9%
Student	38	18.4%
Employee	82	39.6%
Retired	21	10.1%
Do you suffer from any diseases?		
Yes	110	53.1%
No	97	46.9%
Type of disease you have		
Diabetes (DM)	18	16.4%
Hypertension (HTN)	18	16.4%
Unclear / Non-specific entries	6	5.5%
Gastrointestinal (GI) disorders	5	4.5%
Cancer / Tumors	4	3.6%
Respiratory diseases	4	3.6%
Musculoskeletal disorders	4	3.6%
Obesity	3	2.7%
Cardiac conditions	2	1.8%
Gynecological	2	1.8%
General / Chronic disease (unspecified)	2	1.8%
Renal disease	1	0.9%

Slightly more than half of the surgeries were performed in governmental hospitals (*n* = 109; 52.7%). Most patients reported having a companion after surgery (*n* = 149; 72.0%). Considering anesthesia type, general anesthesia was the most frequently used (*n* = 73; 35.3%) (Table 2).

**Table 2:** Surgical data of the study participants (*N* = 207).

Surgery	No	%
Which health agency?		
Governmental hospital	109	52.7%
Private hospital	98	47.3%
Duration since undergoing surgery		
<1 month	32	15.5%
1–3 months	84	40.6%
4–6 months	38	18.4%
>6 months	53	25.6%
Did you have a companion after the operation?		
Yes	149	72.0%
No	58	28.0%
Type of anesthesia or pain control was used during surgery		
General anesthesia	73	35.3%
Local anesthesia	54	26.1%
Spinal/epidural	38	18.4%
Not sure	42	20.3%
Surgical area		
Abdomen	88	43.1%
Pelvis/Gynecology	23	11.3%
Thorax (Chest and Lungs)	25	12.3%
Head and Neck	27	13.2%
Musculoskeletal	25	12.3%
Urology/Renal	4	2.0%
Other/Minor	6	2.9%
Unclear/Miscellaneous	3	1.5%

The majority of participants reported that their healthcare provider discussed pain management options before surgery (*n* = 134; 64.7%), while 73 (35.3%) did not receive such counseling. Regarding concern about postoperative pain, nearly half of the patients were a little worried (*n* = 94; 45.4%). More than half of the participants had been informed about the potential effect of pain on their mobility after surgery (*n* = 122; 58.9%). When asked about their level of preparedness for postoperative pain management, 104 (50.2%) felt fairly prepared (Table 3).

**Table 3:** Preoperative counseling, pain-related concerns, and emotional status of the participants (*N* = 207).

Items	No	%
Did your healthcare provider discuss pain management options with you before surgery?		
Yes	134	64.7%
No	73	35.3%
How concerned were you about feeling pain after surgery?		
Very worried	19	9.2%
Somewhat worried	76	36.7%
A little worried	94	45.4%
Not worried	18	8.7%
Have you been informed about how the pain will affect your ability to move or walk after surgery?		
Yes	122	58.9%
No	85	41.1%
How prepared were you for pain management after surgery?		
Not ready at all	8	3.9%
Very unprepared	68	32.9%
Fairly prepared	104	50.2%
Highly prepared	27	13.0%
How has your mood been for the past 2 weeks?		
Bad	27	13.0%
Good	91	44.0%
I do not know	89	43.0%

On the other hands, the majority of patients reported feeling pain within hours after surgery (*n* = 138; 66.7%). Regarding pain severity within the first 24 h post-surgery, nearly half of the patients (*n* = 91; 46.4%) described their pain as severe (8–10). After 1 week, the intensity of pain appeared to decline, with 101 (51.5%) still experiencing moderate pain. Half of the participants were given pain medication immediately after surgery (*n* = 99; 50.5%). About one-third of the participants (*n* = 70; 33.8%) reported experiencing side effects from pain medications, such as nausea, drowsiness, or constipation. A large majority were prescribed pain medication for home use (*n* = 159; 76.8%), and most of them understood how and when to take it (*n* = 130; 81.8%). In terms of effectiveness, 92 (57.9%) of patients rated their home pain medication as fairly effective, 34 (21.4%) (Table 4).

**Table 4:** Postoperative pain experience, severity, and management among the participants (*N* = 207).

Pain	No	%
When did you feel pain after surgery?		
I do not feel any pain	11	5.3%
Immediately	25	12.1%
Within hours	138	66.7%
Within a day	33	15.9%
Severity of pain after surgery within 24 h after surgery on a scale of 1–10		
Mild (1–3)	23	11.7%
Moderate (4–7)	82	41.8%
Severe (8–10)	91	46.4%
Severity of pain after surgery within 1 week after surgery on a scale of 1 to 10		
Mild (1–3)	58	29.6%
Moderate (4–7)	101	51.5%
Severe (8–10)	37	18.9%
Were you given any pain medication immediately after surgery?		
Yes	99	50.5%
No	64	32.7%
I do not know	33	16.8%
Have you experienced any side effects from pain medications (such as nausea, drowsiness, constipation, stomach pain)?		
Yes	70	33.8%
No	137	66.2%
Have you been prescribed pain medication to take at home?		
Yes	159	76.8%
No	48	23.2%
Did you understand how and when to take pain medications?		
Yes	130	81.8%
No	29	18.2%
How effective was the home pain medication?		
Not effective	23	14.5%
Fairly effective	92	57.9%
Very effective	34	21.4%
I did not have it	10	6.3%

Considering mobility, most patients reported that pain had a small effect on walking or moving independently (*n* = 84; 42.9%). Similarly, postoperative pain was found to influence sleep patterns, with nearly half of the respondents indicating a little effect (*n* = 94; 48.0%). Regarding social interactions, pain had no effect of social interactions (*n* = 67; 34.2%). When asked about the impact of pain on performing daily activities, 69 (35.8%) reported a little effect. Concerning recovery, over one-third of patients regained normal mobility within 7–14 days (*n* = 68; 32.9%) (Table 5).

**Table 5:** Impact of postoperative pain and recovery time among the participants (*N* = 207).

Items	No	%
Effect of pain on walking or moving independently		
No effect at all	55	28.1%
Little effect	84	42.9%
Moderate effect	39	19.9%
Strong effect	18	9.2%
Effect of pain on sleep		
No effect at all	52	26.5%
Little effect	94	48.0%
Moderate effect	36	18.4%
Strong effect	14	7.1%
Effect of pain on interaction with others		
No effect at all	67	34.2%
Little effect	67	34.2%
Moderate effect	47	24.0%
Strong effect	15	7.7%
Effect of pain on performing daily activities		
No effect at all	51	26.4%
Little effect	69	35.8%
Moderate effect	36	18.7%
Strong effect	37	19.2%
How long did it take for you to return to normal mobility		
Less than 3 days	55	26.6%
4–7 days	58	28.0%
7–14 days	68	32.9%
>14 days	26	12.6%

The majority of participants reported being able to communicate their pain levels to healthcare providers to some extent (n = 108; 52.2%). Regarding participation in anesthesia-related decisions, only 74 (35.7%) of the patients indicated that they were involved in choosing the type of anesthesia. Similarly, when asked about involvement in decisions related to analgesic (pain medication) selection, just 65 (31.4%) reported being included in the decision (Table 6).

**Table 6:** Patient communication and involvement in pain management decisions among the participants (*N* = 207).

Items	No	%
Were you able to easily communicate your level of pain to the nurses or doctors?		
Yes	67	32.4%
To some extent	108	52.2%
No	32	15.5%
Were you involved in making decisions about your pain management plan (type of anesthesia)?		
Yes, I participated in choosing the type of anesthesia.	74	35.7%
No, I did not participate	133	64.3%
Were you involved in making decisions about your pain management plan (analgesic)?		
Yes, I participated in choosing the analgesic	65	31.4%
No, I did not participate	142	68.6%

Only 49 (25.0%) of the participants reported using any form of non-pharmacological pain management. Among those who used non-drug techniques, reported the different methods used (*n* = 49; 25%), while (*n* = 149; 75%) did not (Table 7).

**Table 7:** Use of non-drug pain relief techniques among the participants (*N* = 207).

Items	No	%
Have you used any non-drug pain relief techniques (such as ice, physical therapy, relaxation)?		
Yes	49	25.0%
Not reported	147	75.0%
Types of non-drug pain relief techniques		
Relaxation/Breathing/Rest	8	3.9%
Physical therapy/Exercise	4	2.0%
Massage/Support devices	2	1.0%
Walking/Light activity	2	1.0%
Heat/Cold therapy	3	1.5%
Herbal/Alternative remedies	1	0.5%
Other	4	2.0%
Not reported	38	18.4%

**Figure 1:**
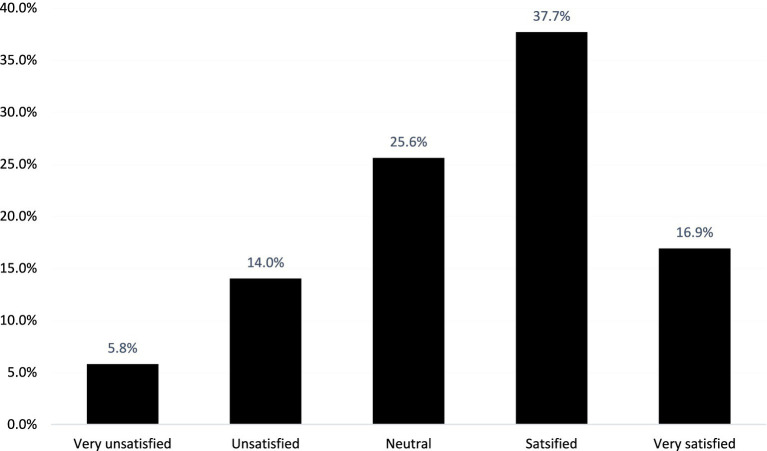
Overall patient satisfaction with postoperative pain management among the participants (*N* = 207).

The majority of participants expressed a positive level of satisfaction, with 78 (37.7%) reporting being satisfied (Figure 1).

**Figure 2:**
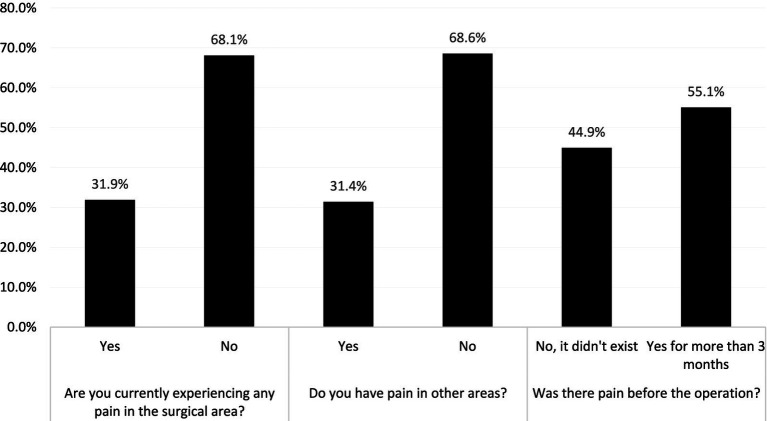
Current and preoperative pain experiences among the participants (*N* = 207).

At the time of the study, 66 (31.9%) participants reported experiencing pain in the surgical area. Similarly, pain in other areas was reported by 65 (31.4%) of patients, Regarding preoperative pain, more than half of the participants (114; 55.1%) had experienced pain for more than 3 months before surgery (Figure 2).

**Figure 3:**
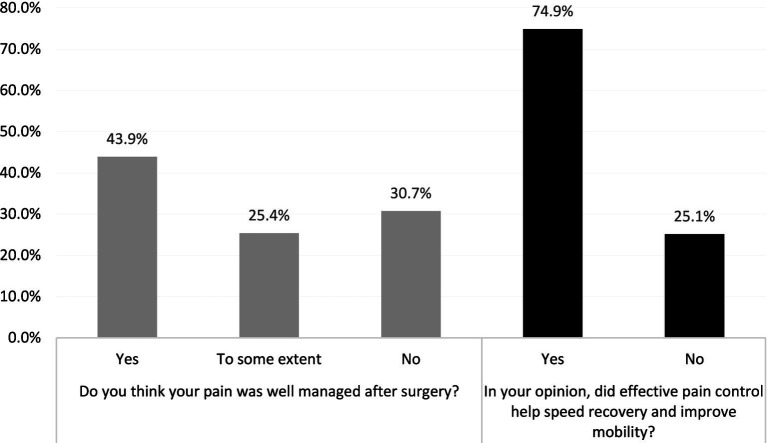
Patient perception of postoperative pain management and its effect on recovery and mobility (*N* = 207).

Less than half of the participants (*n* = 90; 43.9%) felt that their pain was well managed. Despite this, a substantial majority of patients (*n* = 155; 74.9%) perceived that effective pain control contributed to faster recovery and improved mobility (Figure 3).

Regarding factors associated with participants’ satisfaction with pain management after surgery, marital status was significant (*p* = 0.001), with 70.0% of single participants reporting satisfaction. Saudi patients reported significant satisfaction 63.9% (*p* = 0.001). The type of healthcare facility was also important (*p* = 0.001), with 69.7% of patients in governmental hospitals satisfied. The type of anesthesia used showed significance (*p* = 0.001); 74.0% of patients who received general anesthesia were satisfied. Receiving preoperative information about pain medications and non-drug options significantly influenced satisfaction (*p* = 0.001), with 74.4% of those informed about both options reporting to be satisfied. Similarly, discussion of pain management options before surgery was significant (*p* = 0.010), with 61.9% of patients who had a discussion being satisfied. Preparedness for pain management was another significant factor (*p* = 0.004); satisfaction was highest (81.5%) among those highly prepared. Pain severity 1 week after surgery also mattered (*p* = 0.034), with 62.4% of patients with moderate pain satisfied compared to 32.4% with severe pain. Immediate administration of pain medication after surgery influenced satisfaction (*p* = 0.001), with 76.8% of those who received medication immediately satisfied. The ability to communicate pain to nurses or doctors was significant (*p* = 0.004); 73.1% of patients who communicated easily were satisfied. Participation in decisions about anesthesia (*p* = 0.048) also affected satisfaction, with 60.9% satisfaction among those not involved (Table 8).

**Table 8:** Factors associated with participants’ satisfaction with pain management after surgery.

Factors		Overall, how satisfied are you with your pain management?						*p*-value
		Unsatisfied		Neutral		Satisfied		
		No	%	No	%	No	%	
Age	18–29	16	22.5%	18	25.4%	37	52.1%	0.324
	30–49	14	14.1%	27	27.3%	58	58.6%	
	More then 50	11	29.7%	8	21.6%	18	48.6%	
Gender	Male	20	23.5%	19	22.4%	46	54.1%	0.447
	Female	21	17.2%	34	27.9%	67	54.9%	
Marital status	Single	5	10.0%	10	20.0%	35	70.0%	0.001*
	Married	17	16.3%	23	22.1%	64	61.5%	
	Divorced/widow	19	35.8%	20	37.7%	14	26.4%	
Nationality	Saudi	21	14.6%	31	21.5%	92	63.9%	0.001*
	Non-Saudi	20	31.7%	22	34.9%	21	33.3%	
Educational level	Basic education	3	9.7%	10	32.3%	18	58.1%	0.141
	Diplôme, University	25	18.9%	30	22.7%	77	58.3%	
	Post-graduate	13	29.5%	13	29.5%	18	40.9%	
Which health agency?	Governmental hospital	10	9.2%	23	21.1%	76	69.7%	0.001*
	Private hospital	31	31.6%	30	30.6%	37	37.8%	
Do you suffer from any diseases?	Yes	24	21.8%	29	26.4%	57	51.8%	0.650
	No	17	17.5%	24	24.7%	56	57.7%	
Type of anesthesia or pain control was used during surgery	General anesthesia	5	6.8%	14	19.2%	54	74.0%	0.001*
	Local anesthesia	18	33.3%	17	31.5%	19	35.2%	
	Spinal/epidural	12	31.6%	11	28.9%	15	39.5%	
	Not sure	6	14.3%	11	26.2%	25	59.5%	
Did you receive information about pain medications or non-drug pain relief options before surgery?	For both	9	11.5%	11	14.1%	58	74.4%	0.001*
	For medications	13	30.2%	12	27.9%	18	41.9%	
	Only non-drug options	15	28.3%	22	41.5%	16	30.2%	
	No	4	12.1%	8	24.2%	21	63.6%	
Did your healthcare provider discuss pain management options with you before surgery?	Yes	20	14.9%	31	23.1%	83	61.9%	0.010*
	No	21	28.8%	22	30.1%	30	41.1%	
Have you been informed about how the pain will affect your ability to move or walk after surgery?	Yes	20	16.4%	29	23.8%	73	59.8%	0.162
	No	21	24.7%	24	28.2%	40	47.1%	
How prepared were you for pain management after surgery?	Not ready at all	2	25.0%	3	37.5%	3	37.5%	0.004*
	Very unprepared	14	20.6%	27	39.7%	27	39.7%	
	Fairly prepared	21	20.2%	22	21.2%	61	58.7%	
	Highly prepared	4	14.8%	1	3.7%	22	81.5%	
Severity of pain after surgery within 24 h after surgery on a scale of 1–10	Mild (1–3)	5	21.7%	8	34.8%	10	43.5%	0.191
	Moderate (4–7)	19	23.2%	24	29.3%	39	47.6%	
	Severe (8–10)	13	14.3%	20	22.0%	58	63.7%	
Severity of pain after surgery within 1 week after surgery on a scale of 1–10	Mild (1–3)	12	20.7%	14	24.1%	32	55.2%	0.034*
	Moderate (4–7)	14	13.9%	24	23.8%	63	62.4%	
	Severe (8–10)	11	29.7%	14	37.8%	12	32.4%	
Were you given any pain medication immediately after surgery?	Yes	3	3.0%	20	20.2%	76	76.8%	0.001*
	No	22	34.4%	24	37.5%	18	28.1%	
	I do not know	12	36.4%	8	24.2%	13	39.4%	
Were you able to easily communicate your level of pain to the nurses or doctors?	Yes	6	9.0%	12	17.9%	49	73.1%	0.004*
	To some extent	25	23.1%	33	30.6%	50	46.3%	
	No	10	31.3%	8	25.0%	14	43.8%	
Were you involved in making decisions about your pain management plan (type of anesthesia)?	Yes, I participated in choosing the type of anesthesia	19	25.7%	23	31.1%	32	43.2%	0.048*
	No, I did not participate	22	16.5%	30	22.6%	81	60.9%	
Were you involved in making decisions about your pain management plan (analgesic)?	Yes, I participated in choosing the analgesic	15	23.1%	19	29.2%	31	47.7%	0.402
	No, I did not participate	26	18.3%	34	23.9%	82	57.7%	
Have you been prescribed pain medication to take at home?	Yes	24	15.1%	40	25.2%	95	59.7%	0.004*
	No	17	35.4%	13	27.1%	18	37.5%	
How effective was the home pain medication?	Not effective	13	56.5%	6	26.1%	4	17.4%	0.001*
	Fairly effective	8	8.7%	20	21.7%	64	69.6%	
	Very effective	1	2.9%	10	29.4%	23	67.6%	
	I did not have it	2	20.0%	4	40.0%	4	40.0%	
Do you think your pain was well-managed after surgery?	Yes	17	18.9%	20	22.2%	53	58.9%	0.003*
	To some extent	4	7.7%	11	21.2%	37	71.2%	
	No	19	30.2%	21	33.3%	23	36.5%	

P, Pearson *X*^2^ test; ^, Exact probability test.

*p < 0.05 (significant).

## Discussion

The present study assessed the effect of postoperative pain management on patient suffering and mobility among individuals who underwent major surgery in the Makkah region, Saudi Arabia. Our findings indicate that effective pain control significantly influences patient satisfaction, recovery, and functional outcomes, highlighting the importance of structured pain management protocols in surgical care.

The majority of patients were aged 30–49 years, with a greater number of female participants. More than half of the sample reported chronic comorbidities, most commonly diabetes and hypertension. These baseline characteristics are consistent with previous studies indicating that middle-aged adults undergoing major surgery often present with comorbid conditions that may complicate pain experiences and recovery (1, 16). As for preoperative counseling and preparedness, about two-thirds of the patients received preoperative counseling regarding pain management. Nearly half of the participants were moderately concerned about postoperative pain, but only a minority felt highly unprepared. Evidence indicates that preoperative education significantly enhances patients’ coping ability and reduces postoperative anxiety, ultimately improving pain outcomes (17, 18). Our results align with studies showing that structured preoperative counseling increases patient satisfaction and enhances adherence to analgesic regimens (17, 19). Importantly, patients who were well-informed about pain management and non-drug strategies reported higher satisfaction levels (74.4%), supporting the role of patient education in optimizing postoperative care.

As for postoperative pain experiences, consistent with previous literature, most patients experienced pain within hours of surgery, with the highest intensity reported during the first 24 h. Severe pain was reported by about half of the patients; this number gradually declined after 1 week. These findings support the literature indicating that acute postoperative pain peaks in the early postoperative period and diminishes with time and appropriate analgesic interventions (7, 20). Half of the patients received immediate postoperative pain medication, reflecting standard clinical practice aimed at preventing uncontrolled pain and promoting early mobilization (21). Exactly one-third reported side effects, such as nausea or drowsiness, consistent with the documented adverse effect profile of commonly used analgesics (22).

The present study also revealed that the postoperative pain significantly influenced patients’ functional abilities, sleep, social interactions, and recovery time. While most participants reported only minor impairment in mobility, about one-fifth experienced moderate to severe limitations. These observations are consistent with prior studies revealing that uncontrolled postoperative pain impedes ambulation, delays rehabilitation, and prolongs hospital stay (1, 23). Significantly, the majority of patients regained independent mobility within 4–14 days, supporting the hypothesis that effective pain management accelerates functional recovery and enhances patient autonomy (1). Moreover, the vast majority of patients perceived that adequate pain control facilitated faster recovery, assessing the clinical relevance of optimizing analgesic protocols to improve postoperative outcomes.

Effective communication regarding pain and participation in decision-making is also a critical determinant of patient satisfaction. In our study, approximately half of the patients were able to communicate their pain levels to healthcare providers to some extent, while only a minority actively participated in decisions regarding anesthesia and analgesic selection. Previous studies show that patient involvement in pain management improves adherence to treatment, satisfaction, and perceived control over recovery (24, 25). Interestingly, our findings revealed that patients who communicated pain easily reported higher satisfaction, whereas involvement in anesthesia decisions did not consistently correlate with satisfaction, indicating that communication quality may outweigh formal decision-making participation in influencing perceptions of care.

Additionally, only one-quarter of patients utilized non-drug pain relief methods, such as relaxation, physical therapy, or heat/cold therapy. While pharmacologic interventions remain the backbone for acute postoperative pain, evidence supports the adjunctive benefit of non-pharmacological strategies in reducing pain perception and improving mobility (26–28). The low uptake observed may reflect limited awareness or availability of such modalities in the study setting, suggesting an area for targeted intervention.

Concerning patient satisfaction and its determinants, more than half of the patients reported being satisfied or very satisfied with pain management, with factors such as marital status, nationality, type of hospital, anesthesia modality, preoperative counseling, pain severity, immediate analgesia, communication ability, and perceived effectiveness of medication significantly influencing satisfaction. These findings are in line with international literature reporting that patient satisfaction with postoperative pain care is multifactorial, involving clinical, psychological, and organizational aspects (29, 30). The higher satisfaction among patients in governmental hospitals may reflect differences in institutional protocols, staff training, or analgesic availability.

## Conclusion and recommendations

In conclusion, effective postoperative pain management in patients undergoing major surgery in Makkah significantly reduces suffering and improves mobility. Timely analgesic administration, clear home instructions, and preoperative counseling enhanced recovery, independent ambulation, and patient satisfaction. Despite generally positive outcomes, non-pharmacological methods were underused, and some patients continued to experience moderate to severe pain. Satisfaction was influenced by marital status, nationality, hospital type, anesthesia, and perceived effectiveness of analgesics. Recommendations include strengthening preoperative education, promoting multimodal pain management, improving patient-provider communication, and implementing standardized pain management protocols to optimize recovery and reduce postoperative discomfort.

## Data Availability

The original contributions presented in the study are included in the article/supplementary material, further inquiries can be directed to the corresponding author.
